# Metabolic Effects of Gastrectomy and Duodenal Bypass in Early Gastric Cancer Patients with T2DM: A Prospective Single-Center Cohort Study

**DOI:** 10.3390/jcm10174008

**Published:** 2021-09-04

**Authors:** Young Ki Lee, Eun Kyung Lee, You Jin Lee, Bang Wool Eom, Hong Man Yoon, Young-Il Kim, Soo Jeong Cho, Jong Yeul Lee, Chan Gyoo Kim, Sun-Young Kong, Min Kyong Yoo, Yul Hwangbo, Young-Woo Kim, Il Ju Choi, Hak Jin Kim, Mi Hyang Kwak, Keun Won Ryu

**Affiliations:** 1 Division of Endocrinology and Metabolism, Department of Internal Medicine, National Cancer Center, Goyang 10408, Korea; yklee@ncc.re.kr (Y.K.L.); eklee@ncc.re.kr (E.K.L.); yulhwangbo@ncc.re.kr (Y.H.); 2 Department of Cancer Biomedical Science, National Cancer Center Graduate School of Cancer Science and Policy, National Cancer Center, Goyang 10408, Korea; red10000@ncc.re.kr (H.M.Y.); 11996@ncc.re.kr (Y.-I.K.); ksy@ncc.re.kr (S.-Y.K.); 3 Center for Gastric Cancer, National Cancer Center, Goyang 10408, Korea; kneeling79@ncc.re.kr (B.W.E.); crystal5@snu.ac.kr (S.J.C.); jylee@ncc.re.kr (J.Y.L.); glse@ncc.re.kr (C.G.K.); youngwookim@ncc.re.kr (Y.-W.K.); cij1224@ncc.re.kr (I.J.C.); 4 Department of Laboratory Medicine, National Cancer Center, Goyang 10408, Korea; 5 Department of Clinical Nutrition, National Cancer Center, Goyang 10408, Korea; mkyoo52@ncc.re.kr; 6 Department of Cancer Control and Population Health, National Cancer Center Graduate School of Cancer Science and Policy, National Cancer Center, Goyang 10408, Korea; 7 Division of Cardiology, Department of Internal Medicine, National Cancer Center, Goyang 10408, Korea; drkhj@ncc.re.kr (H.J.K.); cardiokmh@ncc.re.kr (M.H.K.)

**Keywords:** gastrectomy, endoscopic submucosal dissection, early gastric cancer, type 2 diabetes mellitus, glycemic control, insulin resistance

## Abstract

We evaluated the metabolic effects of gastrectomies and endoscopic submucosal dissections (ESDs) in early gastric cancer (EGC) patients with type 2 diabetes mellitus (T2DM). Forty-one EGC patients with T2DM undergoing gastrectomy or ESD were prospectively evaluated. Metabolic parameters in the patients who underwent gastrectomy with and without a duodenal bypass (groups 1 and 2, *n* = 24 and *n* = 5, respectively) were compared with those in patients who underwent ESD (control, *n* = 12). After 1 year, the proportions of improved/equivocal/worsened glycemic control were 62.5%/29.2%/8.3% in group 1, 40.0%/60.0%/0.0% in group 2, and 16.7%/50.0%/33.3% in the controls, respectively (*p* = 0.046). The multivariable ordered logistic regression analysis results showed that both groups had better 1-year glycemic control. Groups 1 and 2 showed a significant reduction in postprandial glucose (−97.9 and −67.8 mg/dL), body mass index (−2.1 and −2.3 kg/m^2^), and glycosylated hemoglobin (group 1 only, −0.5% point) (all *p* < 0.05). Furthermore, improvements in group 1 were more prominent when preoperative leptin levels were high (*p* for interaction < 0.05). Metabolic improvements in both groups were also observed for insulin resistance, leptin, plasminogen activator inhibitor-1, and resistin. Gastrectomy improved glycemic control and various metabolic parameters in EGC patients with T2DM. Patients with high leptin levels may experience greater metabolic benefits from gastrectomy with duodenal bypass.

## 1. Introduction

Gastric cancer is the most frequently diagnosed cancer in Korea and has the fifth-highest incidence among newly diagnosed cancer cases worldwide [1,2]. While the incidence of gastric cancer has steadily decreased, the number of gastric cancer survivors has increased due to early diagnosis and improved treatment techniques [1,2,3]. In Korea, the 5-year survival rate of gastric cancer has dramatically improved from 43.9% in 1993–1995 to 76.5% in 2013–2017, and the number of gastric cancer survivors reached about 300,000 in 2017 [1].

Type 2 diabetes mellitus (T2DM) is one of the most common comorbidities that determine overall mortality, non-cancer mortality, and quality of life in cancer survivors [4,5,6]. The prevalence of T2DM has been increasing worldwide, and it reached 13.8% in 2018 in Korea [7,8]. Patients with T2DM are at a higher risk for gastric cancer development, and the incidence of T2DM increases after gastric cancer development [9,10]. Therefore, proper management of T2DM is an important issue in many gastric cancer patients.

Gastrectomy and endoscopic submucosal dissection (ESD) are two curative treatment modalities for early gastric cancer (EGC) that show comparable overall and disease-specific survival [11]. Interestingly, gastrectomy performed as bariatric surgery improves glycemic control in morbidly obese patients with T2DM [12,13,14]. Moreover, studies have reported improvement in T2DM in gastric cancer patients after gastrectomy [15,16,17,18]. This evidence suggests that gastrectomy may have additional benefits over ESD in improving glycemic control in EGC patients with T2DM. However, to date, no study has compared the effects of gastrectomy with those of ESD on glycemic control in gastric cancer patients with T2DM using laboratory results.

This study aimed to prospectively examine the metabolic effects of gastrectomy with or without the duodenal bypass and compare the findings with those for ESD in EGC patients with T2DM. We also aimed to explore preoperative conditions in which the metabolic advantage of gastrectomy over ESD increases to identify patients who would benefit the most from gastrectomy.

## 2. Materials and Methods

### 2.1. Study Subjects and Protocols

This nonrandomized, controlled, prospective cohort study initially recruited 62 eligible EGC patients with T2DM who were scheduled to undergo ESD or gastrectomy between April 2012 and December 2014 at the National Cancer Center in Korea (clinicaltrials.gov accesed on 14 July 2014, identifier: NCT01643811). The enrollment criteria were as follows: (1) histologically proven primary gastric adenocarcinoma; (2) in clinical stage Ia or Ib examined with endoscopy, endoscopic ultrasound, and computed tomography; (3) aged 20–80 years; (4) performance status of 0 or 1 on the Eastern Cooperative Oncology Group scale; (5) diagnosis of T2DM; (6) plan to undergo gastrectomy or ESD; and (7) provision of written informed consent. The exclusion criteria were as follows: (1) having a high risk regarding the operation, such as severe heart disease or respiratory disease; (2) being pregnant or planning for pregnancy; (3) having experienced previous abdominal surgery or radiation therapy; or (4) having a proven more advanced disease than pathological stage II requiring adjuvant chemotherapy.

All treatment options were chosen at the discretion of each surgeon. We categorized all patients into three groups according to the intervention: (1) gastrectomy with duodenal bypass group (total and subtotal gastrectomy with Roux-en-Y gastrojejunostomy, and subtotal gastrectomy with loop gastrojejunostomy), (2) gastrectomy without bypass group (subtotal gastrectomy with gastroduodenostomy), and (3) ESD group (the control). Each preoperative and follow-up (3 and 12 months after treatment) examination included measurements of the patient’s height and body weight, along with blood tests (glycosylated hemoglobin (HbA1c), fasting blood glucose (FBG), 2-hour postprandial glucose (PP2), metabolic hormones, and adipokines). After the follow-up examination at 12 months, the patients were followed up regularly in a routine care setting. The protocol and data were approved by the institutional review board of the National Cancer Center (IRB No. NCCNCS-12-563) and all patients provided written informed consent.

### 2.2. Identification and Management Protocols for T2DM

Patients who had previously received antidiabetic drugs were classified as having diabetes. Among patients with no previous history of diabetes, DM was defined based on the result of preoperative evaluation according to the American Diabetes Association criteria: FBG ≥ 126 mg/dL, random glucose ≥ 200 mg/dL, or HbA1c ≥ 6.5% [19]. During a follow-up after 1 year, the diabetes medications were titrated by endocrinologists to achieve HbA1c < 7.0%.

### 2.3. Metabolic Hormones and Adipokines Measurement

Patient blood samples (fasting and postprandial) were stored in a −70 °C deep freezer and used for the measurement of metabolic hormones and adipokines using Bio-Plex Pro™ Diabetes Assay Panels (Luminex, Austin, TX, USA). Insulin, glucagon, ghrelin, gastric inhibitory polypeptide (GIP), glucagon-like peptide-1 (GLP-1), leptin, plasminogen activator inhibitor-1 (PAI-1), resistin, and visfatin levels were assessed. The homeostasis model of insulin resistance (HOMA-IR) was calculated using the following formula: fasting insulin (IU/mL) × FBG (mg/dL)/405.

### 2.4. Glycemic Control Status Assessment

Glycemic control status was assessed at the 1-year visit. Glycemic control status was considered to be “improved” if patients had lower HbA1c with medication with a dose equal to or lower than the baseline and “worsened” if patients had higher HbA1c with medication with a dose equal to or higher than the baseline. Other cases excluded from the “improved” and “worsened” categories were defined as “equivocal”.

### 2.5. Long-Term Outcomes

The composite event was recorded until 3 February 2021, and it included the recurrence of gastric cancer, myocardial infarction, stroke, coronary revascularization, and all-cause death.

### 2.6. Statistical Analysis

Continuous values were presented as means with standard deviations or medians with interquartile ranges. Categorical values were presented as frequencies and percentages. Baseline characteristics, according to intervention groups, were compared via an analysis of variance followed by a Bonferroni post hoc test, a Kruskal–Wallis test followed by Dunn’s post hoc test, or Fisher’s exact test, according to the variable type.

The association between the types of the intervention and glycemic control status (the order of “improved”, “equivocal”, and “worsened”) at the 1-year visit was assessed using the ordered logistic regression analysis. Demographic characteristics and baseline metabolic parameters were considered as potential confounders, and the final multivariable model was adjusted for statistically significant potential confounders through a stepwise selection method. The associations are presented as odds ratios (ORs) with 95% confidence intervals (CIs).

Each metabolic parameter (HbA1c, FBG, PP2, BMI, and HOMA-IR) and levels of metabolic hormones and adipokines during the 1-year follow-up period were compared between the groups of gastrectomy with duodenal bypass patients, gastrectomy without duodenal bypass patients, and ESD patients using the linear mixed model. HOMA-IR, metabolic hormones, and adipokines levels were log-transformed to improve the normality. The differences between groups were adjusted for age, sex, time from the baseline, and the baseline measurements of each assessed variable. The difference between the groups in log-transformed levels of metabolic hormones and adipokines was exponentially transformed and interpreted as a ratio of hormone levels between the groups on the basis of the following equation:

*difference between groups in log(measurements) = log(measurements in the gastrectomy group) − log(measurements in the ESD group) = log(measurements in the gastrectomy group/measurements in the ESD group) = log(a ratio of measurements between groups).*

Additionally, the changes in each measurement at the 3-month and 1-year visits, relative to the baseline levels, were assessed using a paired *t*-test, and *p*-values were adjusted using Dunnett’s method for multiple comparisons between two visit points and the baseline.

For long-term outcomes, the Kaplan–Meier method was used to generate survival curves, while the log-rank test was performed to evaluate differences in composite event-free survival according to the types of the interventions.

### 2.7. Assessment of Effect Modification

Whether the effects of gastrectomy on the 1-year glycemic control status (with ESD as the control) were altered by the baseline metabolic characteristics was explored using the interaction terms, which were defined as the product of the type of interventions and the levels of each parameter. The significance of the effect modification was tested by entering each interaction term into the multivariable ordered logistic regression model for the 1-year glycemic control status.

Patient subgroups were classified based on the median values of the significant effect modifiers detected in the preceding test. Stratified analyses for changes in metabolic parameters were performed according to the subgroups using the linear mixed models. The significance of the heterogeneity according to the subgroups was tested by entering the product of the type of interventions and subgroups into the linear mixed models.

All statistical analyses were performed using SAS 9.4 (SAS Institute Inc., Cary, NC, USA). Analysis items with *p* < 0.05 were considered to be statistically significant.

## 3. Results

### 3.1. Patient Baseline Characteristics

**Figure 1:**
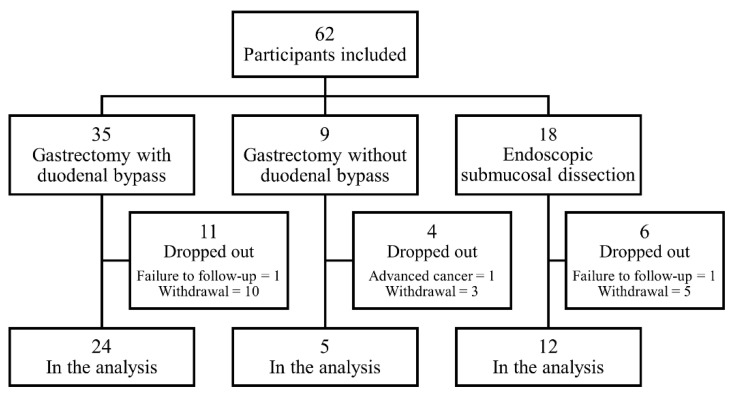
Flow chart of the study.

A total of 62 EGC patients with T2DM were initially enrolled and underwent either gastrectomy or ESD (Figure 1). Among them, 21 patients were excluded due to withdrawal of agreement (*n* = 18), failure to follow-up (*n* = 2), and advancement of the disease beyond pathological stage II (*n* = 1). Finally, a total of 41 patients were included in the 1-year outcome analysis. The number of patients was 24, 5, and 12 in the gastrectomy with duodenal bypass, gastrectomy without duodenal bypass, and ESD groups, respectively. The gastrectomy with duodenal bypass group consisted of patients who underwent a total (*n* = 5) and subtotal (*n* = 9) gastrectomy with Roux-en-Y gastrojejunostomy and patients who underwent subtotal gastrectomy with loop gastrojejunostomy (*n* = 10).

The patient baseline characteristics are presented in Table 1. There were no significant differences in age, sex, duration of diabetes, HbA1c, FBG, PP2, and HOMA-IR between the groups. The mean BMI values were different between the groups (24.1, 21.9, and 26.1 kg/m^2^ in gastrectomy with duodenal bypass, without bypass, and ESD groups, respectively, *p* = 0.022), and the gastrectomy without duodenal bypass group had a lower BMI than did the ESD group (adjusted *p* = 0.024). The levels of metabolic hormones and adipokines were similar between the groups, except for fasting and postprandial PAI-1 levels; the postprandial PAI-1 levels were lower in the gastrectomy without duodenal bypass group than in the ESD group (adjusted *p* = 0.015). Most patients (40 of 41) did not use insulin; the gastrectomy with duodenal bypass group included one patient who took insulin.

### 3.2. Glycemic Control Status at the 1-Year Visit

After 1 year of follow-up, the glycemic control status was different according to the type of intervention (Supplementary Figure S1 online); the proportions of improved/equivocal/worsened glycemic control were 62.5%/29.2%/8.3% in the gastrectomy with duodenal bypass group, 40.0%/60.0%/0.0% in the gastrectomy without duodenal bypass group, and 16.7%/50.0%/33.3% in the ESD group, respectively (*p* = 0.046).

The independent effect of each type of surgery on the 1-year glycemic control status was assessed using ordered logistic regression analysis (Table 2). In the univariable analysis, gastrectomy with duodenal bypass was associated with a better glycemic control status than was an ESD (OR = 7.93, 95% CI = 1.81–34.70). In the final multivariable model, the effects of gastrectomy were adjusted for the baseline HOMA-IR, which was the only significant variable among potential confounders, including age, sex, DM duration, BMI, and HbA1c. In this final model, both gastrectomy with duodenal bypass (OR = 8.68, 95% CI = 1.81–41.63) and gastrectomy without duodenal bypass (OR = 10.60, 95% CI = 1.10–102.35) were associated with a better glycemic control status than was ESD. These estimates for ORs were similar in the full multivariable model that included all potential confounding variables.

### 3.3. Changes in Metabolic Parameters after Gastrectomy

To investigate the effect of surgery on the metabolic parameters (HbA1c, FBG, PP2, BMI, and HOMA-IR), we compared each measurement during the follow-up period between the groups (Table 3). Compared with the ESD group, the gastrectomy with duodenal bypass group showed significantly lower HbA1c (−0.5% point), PP2 (−97.9 mg/dL), BMI (−2.1 kg/m^2^), and log10-transformed HOMA-IR (−0.21) (all *p* < 0.05). The gastrectomy without duodenal bypass group showed similar patterns of metabolic improvements in PP2 (−67.8 mg/dL), BMI (−2.3 kg/m^2^), and log10-transformed HOMA-IR (−0.31) (all *p* < 0.05), but the improvement in HbA1c was not significant (−0.5% point, *p* = 0.184). The improvement in FBG was not significant in either of the gastrectomy groups, with or without duodenal bypass.

Metabolic parameters at the 3-month and 1-year visits were also compared with the baseline levels (Table 3 and Supplementary Figure S2 online). Compared with the preoperative levels, HbA1c, PP2, and BMI showed significant improvements in the gastrectomy with duodenal bypass group, while only BMI showed a significant improvement in the gastrectomy without duodenal bypass group. In contrast, the ESD group showed significant worsening in the FBG levels (+23.8 mg/dL at the 1-year visit, *p* = 0.024).

### 3.4. Changes in Metabolic Hormones and Adipokines after Gastrectomy

**Figure 2:**
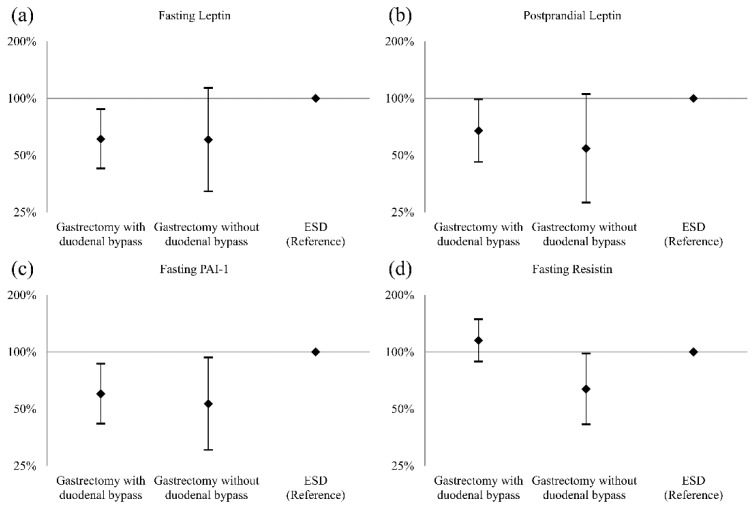
The ratios of metabolic hormone and adipokine levels after gastrectomy and after ESD (the reference). (**a**) Fasting leptin, (**b**) postprandial leptin, (**c**) fasting PAI-1, and (**d**) fasting resistin. The ratios and 95% confidence intervals were estimated using linear mixed models for log-transformed hormone levels, with adjustments for age, sex, time from the baseline, and the baseline measurements of each assessed variable. ESD, endoscopic submucosal dissection; PAI-1, plasminogen activator inhibitor-1.

Metabolic hormone and adipokine levels in the gastrectomy groups during the follow-up period were compared with those in the ESD group (Figure 2 and Supplementary Table S1 online). The gastrectomy with duodenal bypass group showed a significant reduction in fasting leptin, postprandial leptin, and fasting PAI-1 levels (reduced to 61.1%, 67.5%, and 60.1% of those in the ESD group, respectively, all *p* < 0.05). The gastrectomy without duodenal bypass group showed a similar magnitude of reduction in fasting leptin, postprandial leptin, and fasting PAI-1 levels (reduced to 60.5%, 54.4%, and 53.3% of those in the ESD group, respectively), but the reduction in leptin levels was not statistically significant. Fasting resistin levels were reduced only in the gastrectomy without duodenal bypass group (reduced to 63.8% of those in the ESD group, *p* = 0.040). Ghrelin, GIP, GLP-1, glucagon, visfatin, postprandial PAI-1, and postprandial resistin levels after gastrectomy were not different from those in the ESD groups.

### 3.5. Factors Influencing the Metabolic Effects of Gastrectomy with Duodenal Bypass

We explored the influence of preoperative metabolic characteristics on the improvement of the 1-year glycemic control status in patients treated using gastrectomy with duodenal bypass (Supplementary Table S2 online). Due to a small number of cases, potential effect modifiers for gastrectomy without duodenal bypass were not explored. In this exploratory analysis, the beneficial effect of gastrectomy with duodenal bypass was more prominent in patients with higher preoperative fasting or postprandial leptin levels (*p* for interaction = 0.011 and 0.009, respectively) but was attenuated in those with higher preoperative fasting PAI-1 levels (*p* for interaction = 0.013). The effect of gastrectomy with duodenal bypass on the 1-year glycemic control status was not changed by the preoperative BMI, HbA1c, FBG, PP2, HOMA-IR, other metabolic hormones, or adipokines levels.

Next, patient subgroups were classified according to the median values of the preoperative fasting leptin (2.1 ng/mL), postprandial leptin (1.8 ng/mL), and fasting PAI-1 (47.6 ng/mL) levels (Table 4). In the stratified analyses, those with high fasting leptin levels showed a greater decrease in PP2 (−127.1 vs. −72.3 mg/dL, *p* for interaction = 0.017) and BMI (−3.1 vs. −1.2 kg/m^2^, *p* for interaction = 0.018) than did those with lower fasting leptin levels, after gastrectomy with duodenal bypass. Similarly, patients with high postprandial leptin levels showed a greater decrease in PP2 (−133.5 vs. −72.4 mg/dL, *p* for interaction = 0.017) and BMI (−3.0 vs. −1.2 kg/m^2^, *p* for interaction = 0.010) than did those with lower postprandial leptin levels. However, the metabolic improvement after gastrectomy with duodenal bypass was not different, according to fasting PAI-1 levels.

### 3.6. Long-Term Outcomes

During the postoperative follow-up period (median, 5.7 years; interquartile range, 4.9–6.9), one case each of recurrence (ESD group), stroke (gastrectomy without duodenal bypass group), and coronary revascularization (ESD group), and three cases of death from other malignancies (gastrectomy with duodenal bypass group; one biliary cancer and two hematologic malignancies) were recorded (Supplementary Figure S3 online). There was no difference in the composite event-free survival rate between groups (*p* = 0.647).

## 4. Discussion

This single-center prospective controlled cohort study compared standard curative treatment modalities for EGC in patients with T2DM in terms of metabolic effects. EGC patients with T2DM who underwent gastrectomy, with or without duodenal bypass, showed improvement in glycemic control status more frequently than did those who underwent ESD at 1 year postoperatively. The metabolic improvement by gastrectomy was significant in terms of the PP2, HbA1c, and BMI, as well as some metabolic hormones and adipokines, such as leptin and PAI-1. In particular, the patients with higher preoperative leptin levels experienced a greater metabolic benefit from gastrectomy with duodenal bypass versus ESD than did those with lower leptin levels; in this subgroup, the probability for better 1-year glycemic control status was much higher and the degree of improvement in PP2 and BMI was more pronounced.

It is well established in meta-analyses of randomized controlled trials (RCTs) that gastrectomy, performed as bariatric surgery, is excellent at improving or alleviating serum glucose in obese T2DM patients compared to medical therapy [20,21]. In the meta-analysis by Pack et al., both Roux-en-Y gastric bypass and sleeve gastrectomy showed higher remission rates than did standard medical therapy at 1 to 2 years post operation (risk ratios for remission = 9.13 and 11.15, respectively), and this superiority was maintained until 5 years post operation [21]. Bariatric surgery reduced the microvascular and macrovascular diabetic complications and improved the related mortality [22,23,24]. Although most studies included patients with BMI > 35 kg/m^2^, meta-analyses of selected RCTs and non-randomized studies showed that bariatric surgery was similarly effective in T2DM patients with BMI < 30~35 kg/m^2^ [25,26,27].

Gastrectomy to treat gastric cancer is technically similar to bariatric surgery; therefore, it was expected to have metabolic benefits in gastric cancer patients with T2DM. Several studies have discussed improvement in glycemic control and weight reduction after gastrectomy in gastric cancer patients [15,16,17,18,28,29]. However, no studies have compared gastrectomy to non-surgical treatment in gastric cancer patients, except for our previous epidemiological study [18]. This absence of an appropriate control group is an important limitation that can distort the estimate of the effect of gastrectomy in existing observational studies. Previously, we analyzed the Korean National Health Insurance System claims database and showed that, compared with endoscopic resection, total gastrectomy decreased the requirement for antidiabetic medications in gastric cancer patients [18]. However, due to the lack of biochemical data, the improvement of disease control could only be assessed with drug discontinuation [18]. In the current study, we regularly evaluated antidiabetic medications; biochemical data, including serum glucose and HbA1c levels; and anthropometric parameters. Consequently, we showed that the glycemic control status and BMI in EGC patients with T2DM who underwent gastrectomy were significantly improved relative to those in patients who underwent ESD.

In this study, patients who underwent gastrectomy with duodenal bypass had lower leptin and PAI-1 levels than did those who underwent ESD. The improvement in metabolic hormones and adipokines levels after gastrectomy has been demonstrated in studies on bariatric surgery, and Askarpour et al. reported in their recent meta-analysis that bariatric surgery reduced serum leptin, PAI-1, and chemerin levels [30]. A decrease in leptin levels after gastrectomy was also reported in gastric cancer patients, although the control group with non-surgical treatment was limited [15]. Leptin is a satiety hormone that is secreted mainly by the adipocytes [31]. It decreases body weight by suppressing appetite and promoting energy expenditure in physiologic conditions, but hyperleptinemia is observed in patients with obesity and T2DM due to leptin resistance [31,32]. Hyperleptinemia is associated with insulin resistance and micro- and macrovascular diabetic complications, and leptin-mediated hypertension was suggested as one of the mechanisms of developing cardiovascular diseases [32,33,34]. PAI-1 is an inflammatory adipokine that is associated with T2DM, diabetic nephropathy, and cardiovascular diseases [35,36]. Thus, reductions in leptin and PAI-1 levels after gastrectomy in ECG patients with T2DM might predict or mediate a reduction in risk for diabetic complications.

Another notable finding in this study was that higher preoperative leptin levels played a predictive role for a greater metabolic benefit from gastrectomy with duodenal bypass versus ESD. Such a predictive role has not been widely investigated, but there are a few recent studies on this topic [15,37]. In an RCT that included 40 patients that compared the glycemic control effects of gastric cancer surgery according to surgery type, patients who experienced improvement or remission of diabetes at 12 months after surgery had higher preoperative leptin levels than those who did not [15]. In contrast, in a cohort study on 38 obese patients (mean BMI = 47.3 kg/m^2^) with diabetes who underwent bariatric surgery, those with higher than mean preoperative leptin levels (27.3 ng/mL) had higher glucose levels at 3 months post operation [37]. However, this leptin level was approximately 13 times higher than that measured in our study (2.1 ng/mL) due to differences in study populations (obesity vs. EGC) [37]. Since an improvement in hyperleptinemia is one of the remarkable effects of gastrectomy [30], the metabolic benefit from gastrectomy might be less prominent in those without hyperleptinemia. Our results suggest that choosing gastrectomy with duodenal bypass over ESD might be particularly advantageous in EGC patients with T2DM with high leptin levels.

This study had some limitations. The small sample size of this study led to underpowered results, and results on gastrectomy without duodenal bypass may not be reliable due to the small number of patients included. In addition, given the exploratory nature of the study, statistical adjustment for multiplicity was not conducted for multiple outcomes. Therefore, the possibility cannot be excluded that some of the statistically significant results in this study appeared by chance and, thus, they should be interpreted with caution based on the existing scientific knowledge. Because this was a nonrandomized observational study, the results may have been influenced by unmeasured confounders, although most of the measured potential confounders did not differ significantly between groups and were additionally controlled in multivariable analyses. The high dropout rate (33.9%) might serve as a source of bias via differential dropout, although the dropout rates were similar between groups, and the most common reason for dropout was the withdrawal of consent rather than medical problems or failure to follow-up. Furthermore, we could not confirm the difference in the long-term cardiovascular outcome, which could be dependent on diabetes control, due to the small number of events. Studies with a larger sample size are warranted to overcome these limitations and to validate our results.

In summary, our study suggests that gastrectomy has an advantage over ESD in terms of better diabetes management and weight reduction in EGC patients with T2DM and that this advantage can be more prominent in those with higher leptin levels. Metabolic benefits from gastrectomy should be considered in treatment decisions in these patients.

## Figures and Tables

**Table 1:** Baseline characteristics of patients included in the analysis.

Baseline Characteristics	Total (*n* = 41)	Gastrectomy with Duodenal Bypass (*n* = 24)	Gastrectomy without Duodenal Bypass (*n* = 5)	Endoscopic Submucosal Dissection (*n* = 12)	*p*-Value
Age (years)	62.4 ± 8.4	61.6 ± 9.0	63.8 ± 10.9	63.3 ± 6.3	0.800
Female sex	8 (19.5%)	4 (16.7%)	3 (60.0%)	1 (8.3%)	0.054
DM duration (years)	6.8 ± 6.1	6.1 ± 5.7	6.2 ± 3.9	8.6 ± 7.5	0.489
BMI (kg/m^2^)	24.5 ± 3.1	24.1 ± 2.6	21.9 ± 1.7 *	26.1 ± 3.6	**0.022**
HbA1c (%)	7.3 ± 1.6	7.5 ± 1.8	6.8 ± 1.5	7.1 ± 0.9	0.579
Fasting glucose (mg/dL)	132.5 ± 43.7	141.2 ± 53.3	120.8 ± 18.8	120.2 ± 22.3	0.332
Postprandial 2-hour glucose (mg/dL)	292.4 ± 97.8	301.2 ± 105.2	318.8 ± 140.5	262.9 ± 52.5	0.474
HOMA-IR	1.7 (1.1–3.8)	2.5 (1.2–3.8)	1.0 (0.9–1.5)	1.7 (1.3–4.3)	0.169
**Fasting**					
Ghrelin (pg/mL)	501.8 (242.1–935.8)	460.6 (234.7–968.5)	941.5 (152.5–1457.0)	520.5 (372.5–873.8)	0.937
GIP (pg/mL)	211.3 (123.1–261.5)	235.4 (170.6–295.9)	211.2 (118.6–224.7)	144.7 (113.7–204.7)	0.097
GLP-1 (pg/mL)	191.8 (102.9–259.5)	222.5 (134.3–261.8)	129.8 (93.4–538.9)	186.6 (72.9–232.4)	0.657
Glucagon (pg/mL)	124.0 (75.7–258.4)	144.2 (78.7–263.3)	84.8 (79.6–385.3)	85.1 (69.6–121.4)	0.182
Leptin (ng/mL)	2.1 (1.1–3.9)	2.1 (1.1–4.0)	1.2 (0.7–5.8)	2.4 (0.9–3.0)	0.932
PAI-1 (ng/mL)	47.6 (37.5–72.6)	43.5 (35.8–66.9)	35.2 (28.6–71.7)	53.8 (47.6–149.0)	**0.036**
Resistin (ng/mL)	5.4 (3.0–8.8)	5.1 (2.6–8.0)	5.1 (3.5–10.6)	5.9 (3.2–9.5)	0.570
Visfatin (ng/mL)	2.7 (1.1–5.8)	2.7 (0.8–4.6)	4.1 (1.5–9.4)	2.3 (1.3–6.8)	0.406
**Postprandial 2 h**					
Ghrelin (pg/mL)	444.3 (278.3–774.5)	370.7 (273.3–751.5)	693.9 (123.3–1173.3)	505.2 (374.4–779.3)	0.789
GIP (pg/mL)	355.1 (299.8–474.2)	367.4 (297.0–551.3)	427.3 (368.6–496.8)	314.6 (260.0–445.4)	0.418
GLP-1 (pg/mL)	222.3 (127.5–276.9)	229.4 (147.2–291.8)	206.6 (162.1–530.3)	205.6 (85.5–266.6)	0.624
Glucagon (pg/mL)	127.2 (74.8–245.3)	172.7 (83.8–251.3)	110.1 (82.8–382.7)	91.2 (70.8–139.0)	0.453
Leptin (ng/mL)	1.8 (0.9–3.3)	1.7 (0.9–3.4)	1.1 (0.7–5.4)	1.8 (0.9–2.6)	0.972
PAI-1 (ng/mL)	45.1 (33.7–69.0)	47.5 (31.1–66.1)	33.7 (25.6–37.1) *	61.0 (43.2–222.9)	**0.016**
Resistin (ng/mL)	4.5 (2.5–6.5)	4.9 (2.7–6.0)	3.9 (2.5–5.8)	3.8 (2.4–9.4)	0.867
Visfatin (ng/mL)	2.0 (1.0–5.8)	1.9 (0.9–5.2)	2.1 (1.6–4.4)	2.2 (0.9–9.7)	0.762

Data are presented as mean ± standard deviation, median (interquartile range), or frequency (%). Significant *p*-values (*p* < 0.05) are in boldface type. * Significant difference from the ESD group in the post hoc analysis (adjusted *p* < 0.05). DM, diabetes mellitus; BMI, body mass index; HbA1c, hemoglobin A1c; HOMA-IR, homeostasis model assessment of insulin resistance; ESD, endoscopic submucosal dissection; GIP, gastric inhibitory polypeptide; GLP-1, glucagon-like peptide-1; PAI-1, plasminogen activator inhibitor-1.

**Table 2:** The effects of gastrectomy with or without duodenal bypass on the probability of better glycemic control at the 1-year visit.

Variables	Univariable Model		Full Multivariable Model		Final Multivariable Model ^1^	
	OR (95% CI)	*p*-Value	OR (95% CI)	*p*-Value	OR (95% CI)	*p*-Value
Type of the intervention						
ESD	1	Reference	1	Reference	1	Reference
Gastrectomy with duodenal bypass	**7.93 (1.81–34.70)**	**0.006**	**11.94 (2.03–70.26)**	**0.006**	**8.68 (1.81–41.63)**	**0.007**
Gastrectomy without duodenal bypass	4.27 (0.55–33.37)	0.166	12.02 (0.74–193.91)	0.080	**10.60 (1.10–102.35)**	**0.041**
Age (years)	0.98 (0.92–1.06)	0.658	0.99 (0.90–1.09)	0.890		
Female sex	2.05 (0.44–9.54)	0.359	1.98 (0.24–16.18)	0.525		
DM duration (years)	0.92 (0.84–1.02)	0.119	0.97 (0.85–1.11)	0.658		
BMI (kg/m^2^)	1.06 (0.87–1.29)	0.557	1.15 (0.87–1.53)	0.323		
HbA1c (%)	0.90 (0.62–1.30)	0.562	0.79 (0.48–1.31)	0.362		
HOMA-IR	**1.65 (1.07–2.54)**	**0.024**	**1.96 (1.08–3.55)**	**0.027**	**1.88 (1.16–3.07)**	**0.011**

The association between each baseline characteristic, including the type of the intervention and better glycemic control (the order of “improved”, “equivocal”, and “worsened”) at the 1-year visit is presented as an OR and its CI estimated using ordered logistic regression analysis. ^1^ Variables included in the final multivariable model were selected through a stepwise selection method. This model, including only significant variables, was chosen as the final model for parsimoniousness. Significant values (*p* < 0.05) are in boldface type. OR, odds ratio; CI, confidence interval; ESD, endoscopic submucosal dissection; DM, diabetes mellitus; BMI, body mass index; HbA1c, hemoglobin A1c; HOMA-IR, homeostasis model assessment of insulin resistance.

**Table 3:** Metabolic parameters during the 1-year follow-up period.

Metabolic Parameters	Groups	Difference from the Control Group ^1^		Change from the Baseline ^2^			
				3-Month Visit		1-Year Visit	
		Estimates	*p*-Value	Mean ± SD	*p*-Value	Mean ± SD	*p*-Value
HbA1c (%)	ESD	0	Ref	−0.3 ± 1.2	0.885	−0.1 ± 1.5	>0.999
	Group 1	**−0.5**	**0.028**	**−1.1 ± 1.6**	**0.007**	**−0.9 ± 1.7**	**0.045**
	Group 2	−0.5	0.184	−0.5 ± 1.1	0.640	0.0 ± 1.3	>0.999
Fasting glucose (mg/dL)	ESD	0	Ref	17.4 ± 27.1	0.095	**23.8 ± 27.4**	**0.024**
	Group 1	−11.1	0.328	−12.2 ± 71.8	0.831	0.2 ± 54.5	>0.999
	Group 2	−0.6	0.971	19.2 ± 33.2	0.532	9.0 ± 21.6	0.808
Postprandial 2 h glucose (mg/dL)	ESD	0	Ref	−17.2 ± 49.4	0.551	13.6 ± 50.2	0.828
	Group 1	**−97.9**	**<0.001**	**−151.1 ± 103.8**	**<0.001**	**−99.0 ± 109.0**	**0.001**
	Group 2	**−67.8**	**0.044**	−121.4 ± 123.5	0.186	−117.0 ± 113.2	0.261
BMI (kg/m^2^)	ESD	0	Ref	−0.2 ± 1.3	>0.999	−0.4 ± 1.9	0.983
	Group 1	**−2.1**	**<0.001**	**−2.2 ± 1.8**	**<0.001**	**−1.6 ± 1.9**	**0.002**
	Group 2	**−2.3**	**0.001**	**−2.0 ± 0.4**	**0.001**	−1.5 ± 0.8	0.062
Log(HOMA-IR)	ESD	0	Ref	0.00 ± 0.31	>0.999	0.01 ± 0.30	>0.999
	Group 1	**−0.21**	**0.019**	−0.21 ± 0.44	0.064	−0.28 ± 0.58	0.067
	Group 2	**−0.31**	**0.036**	−0.10 ± 0.41	>0.999	0.01 ± 0.11	>0.999

^1^ During the 1-year follow-up period, each metabolic parameter in the gastrectomy with duodenal bypass group (group 1) and the gastrectomy without duodenal bypass group (group 2) was compared with each respective parameter in the control group using a linear mixed model. The estimates and *p*-values were adjusted for age, sex, time from the baseline, and the baseline measurements of each assessed variable. ^2^ Statistical significance of the change in each variable at each visit, relative to the baseline value, was assessed using a paired *t*-test. The *p*-values were adjusted using Dunnett’s method for multiple comparisons between two visit points and the baseline. Significant values (*p* < 0.05) are in boldface type. SD, standard deviation; HbA1c, hemoglobin A1c; ESD, endoscopic submucosal dissection; Ref, reference value; BMI, body mass index; Log, log10-transformed; HOMA-IR, homeostasis model assessment of insulin resistance.

**Table 4:** Changes in the metabolic parameters after gastrectomy with duodenal bypass compared with ESD as a reference in the subgroups classified by preoperative fasting leptin, postprandial leptin, and fasting PAI-1 levels.

Metabolic Parameters	Subgroups by Preoperative Fasting Leptin					Subgroups by Preoperative Postprandial Leptin					Subgroups by Preoperative Fasting PAI-1				
	High (≥2.1 ng/mL)		Low (<2.1 ng/mL)		*p* for Interaction ^1^	High (≥1.8 ng/mL)		Low (<1.8 ng/mL)		*p* for Interaction ^1^	High (≥47.6 ng/mL)		Low (<47.6 ng/mL)		*p* for Interaction ^1^
	Effects	*p*-Value	Effects	*p*-Value		Effects	*p*-Value	Effects	*p*-Value		Effects	*p*-Value	Effects	*p*-Value	
HbA1c (%)	−0.6	0.016	−0.4	0.342	0.182	−0.6	0.011	−0.5	0.229	0.290	−0.3	0.505	−1.1	0.009	0.510
Fasting glucose (mg/dL)	−22.3	0.068	6.9	0.718	0.255	−20.2	0.065	2.6	0.896	0.381	−7.9	0.641	−6.5	0.741	0.574
Postprandial 2-h glucose (mg/dL)	**−127.1**	**<0.001**	**−72.3**	**0.050**	**0.017**	**−133.5**	**<0.001**	**−72.4**	**0.046**	**0.017**	**−80.5**	**0.005**	**−100.3**	**0.002**	0.375
BMI (kg/m^2^)	**−3.1**	**<0.001**	−1.2	0.090	**0.018**	**−3.0**	**<0.001**	−1.2	0.075	**0.010**	**−1.7**	**0.004**	**−3.4**	**<0.001**	0.147
Log(HOMA-IR)	**−0.9**	**0.001**	−0.1	0.743	0.223	**−0.9**	**0.002**	−0.1	0.696	0.224	−0.3	0.322	−0.9	0.015	0.266

The effects of gastrectomy with duodenal bypass on each metabolic parameter during the follow-up period, relative to the effects of ESD (the control), were estimated using a linear mixed model in the subgroups that were classified by preoperative fasting leptin, postprandial leptin, and fasting PAI-1 levels. The estimates and *p*-values were adjusted for gastrectomy without duodenal bypass, age, sex, time from the baseline, and the baseline measurements of each assessed metabolic parameter. ^1^ Significance of the differences in the effects of gastrectomy with duodenal bypass according to the subgroups was tested by entering the product of “gastrectomy with duodenal bypass” and the subgroups into the linear mixed models. Significant values (*p* < 0.05 and *p* for interaction <0.05) are in boldface type. ESD, endoscopic submucosal dissection; PAI-1, plasminogen activator inhibitor-1; HbA1c, hemoglobin A1c; BMI, body mass index; Log, log10-transformed; HOMA-IR, homeostasis model assessment of insulin resistance.

## Data Availability

The datasets generated for this study are available on reasonable request to the corresponding author. The data are not publicly available due to privacy reasons.

## Supplementary Materials

The following are available online at https://www.mdpi.com/article/10.3390/jcm10174008/s1: Supplementary Table S1. Changes in the metabolic hormone levels (log10-transformed); Supplementary Table S2. Changes in the effects of gastrectomy with duodenal bypass according to the candidate effect modifiers on the probability of better 1-year glycemic control at the 1-year visit; Supplementary Figure S1. Glycemic control status at the 1-year visit according to the type of intervention. ESD, endoscopic submucosal dissection; Supplementary Figure S2. Changes in the metabolic parameters from the baseline values; Supplementary Figure S3. Kaplan–Meier curves for composite events (recurrence of gastric cancer, myocardial infarction, stroke, coronary revascularization, and all-cause death) according to the type of intervention.
