# Supplementary material for: Metabolic Effects of Gastrectomy and Duodenal Bypass in Early Gastric Cancer Patients with T2DM: A Prospective Single-Center Cohort Study
Source: J Clin Med. 2021 Sep 4;10(17):4008. doi: 10.3390/jcm10174008 (PMC8432535; doi:10.3390/jcm10174008)
Supplement: Supplementary file 1 [file jcm-10-04008-s001.zip › jcm-1319495-supplementary.pdf]

Supplementary information

Metabolic Effects of Gastrectomy and Duodenal Bypass in Early Gastric Cancer Patients with T2DM: A Prospective Single-Center Cohort Study

Young Ki Lee[1#], Eun Kyung Lee[1,2#], You Jin Lee[1*], Bang Wool Eom[3], Hong Man Yoon, [2,3], Young-Il Kim[2,3], Soo Jeong Cho[3], Jong Yeul Lee[3], Chan Gyoo Kim[3], Sun-Young Kong[2,4], Min Kyong Yoo[5], Yul Hwangbo[1,6], Young-Woo Kim[3], Il Ju Choi[3,6], Hak Jin Kim[7], Mi Hyang Kwak[7] and Keun Won Ryu[3*]

Supplementary Table S1. Changes in the metabolic hormone levels (log10-transformed).

Variables	Groups	Difference compared to the control group[1]			Change from the baseline[2]			
					3-month visit		1-year visit	
		Estimates	10^(estimates)	p value	Mean±SD	p value	Mean±SD	p value
**Fasting**								
Log (ghrelin)	ESD	0	1	Ref.	0.13±0.33	0.410	0.04±0.30	>0.999
	Group 1	0.02	1.05	0.800	0.06±0.40	0.985	0.18±0.41	0.092
	Group 2	-0.07	0.85	0.623	0.13±0.37	>0.999	-0.12±0.23	0.784
Log (GIP)	ESD	0	1	Ref.	0.14±0.21	0.066	0.18±0.52	0.497
	Group 1	-0.06	0.87	0.471	0.06±0.31	0.665	-0.01±0.38	>0.999
	Group 2	-0.01	0.98	0.933	0.13±0.20	0.562	0.01±0.34	>0.999
Log (GLP1)	ESD	0	1	Ref.	0.14±0.30	0.256	0.20±0.48	0.377
	Group 1	0.04	1.10	0.659	0.13±0.36	0.191	0.25±0.51	0.062
	Group 2	-0.03	0.94	0.868	0.01±0.33	>0.999	-0.14±0.55	>0.999
Log (glucagon)	ESD	0	1	Ref.	0.23±0.48	0.322	**0.46±0.54**	**0.027**
	Group 1	-0.01	0.97	0.899	0.09±0.41	0.635	0.14±0.54	0.485
	Group 2	-0.15	0.70	0.441	0.09±0.39	>0.999	-0.10±0.18	0.725
Log (leptin)	ESD	0	1	Ref.	-0.07±0.19	0.432	0.03±0.40	>0.999
	Group 1	**-0.21**	**0.61**	**0.008**	**-0.27±0.30**	**0.001**	-0.17±0.57	0.326
	Group 2	-0.22	0.61	0.116	-0.29±0.53	0.699	-0.06±0.63	>0.999
Log (PAI-1)	ESD	0	1	Ref.	-0.12±0.47	0.910	-0.20±0.90	0.973
	Group 1	**-0.22**	**0.60**	**0.007**	-0.09±0.25	0.189	-0.06±0.37	0.851
	Group 2	**-0.27**	**0.53**	**0.029**	-0.13±0.21	0.618	-0.12±0.31	>0.999
Log (resistin)	ESD	0	1	Ref.	**-0.20±0.25**	**0.035**	-0.18±0.39	0.297
	Group 1	0.06	1.15	0.279	0.00±0.27	>0.999	-0.08±0.37	0.648
	Group 2	**-0.20**	**0.64**	**0.040**	-0.36±0.44	0.394	-0.34±0.29	0.203
Log (visfatin)	ESD	0	1	Ref.	0.06±0.36	>0.999	-0.15±0.67	0.941
	Group 1	-0.04	0.91	0.754	-0.03±0.54	>0.999	0.07±0.54	>0.999
	Group 2	-0.37	0.43	0.107	-0.60±0.87	0.718	-0.29±0.29	0.279
**Postprandial 2 hours**								
Log (ghrelin)	ESD	0	1	Ref.	0.07±0.28	0.790	0.04±0.26	>0.999
	Group 1	0.01	1.02	0.941	0.00±0.40	>0.999	0.20±0.38	0.053

	Group 2	-0.12	0.75	0.445	0.29±0.29	0.288	-0.42±0.69	0.627
Log (GIP)	ESD	0	1	Ref.	0.00±0.27	>0.999	0.26±0.58	0.344
	Group 1	-0.08	0.82	0.291	-0.02±0.30	>0.999	0.02±0.41	>0.999
	Group 2	-0.05	0.90	0.736	0.00±0.17	>0.999	-0.26±0.53	0.816
Log (GLP1)	ESD	0	1	Ref.	0.08±0.33	0.894	0.21±0.53	0.427
	Group 1	0.04	1.10	0.722	0.05±0.48	>0.999	0.24±0.54	0.105
	Group 2	0.14	1.39	0.485	0.11±0.14	0.421	-0.20±0.62	>0.999
Log (glucagon)	ESD	0	1	Ref.	0.19±0.41	0.354	**0.46±0.47**	**0.018**
	Group 1	0.01	1.02	0.944	0.14±0.41	0.268	0.20±0.53	0.231
	Group 2	0.12	1.32	0.582	0.21±0.20	0.262	-0.02±0.39	>0.999
Log (leptin)	ESD	0	1	Ref.	-0.08±0.21	0.475	0.13±0.48	0.792
	Group 1	**-0.17**	**0.68**	**0.043**	**-0.20±0.34**	**0.033**	-0.16±0.47	0.284
	Group 2	-0.26	0.54	0.070	-0.32±0.46	0.517	-0.15±0.54	>0.999
Log (PAI-1)	ESD	0	1	Ref.	-0.28±0.40	0.062	-0.34±0.75	0.326
	Group 1	-0.05	0.90	0.537	-0.07±0.28	0.613	-0.01±0.28	>0.999
	Group 2	-0.11	0.78	0.390	0.02±0.22	>0.999	0.00±0.27	>0.999
Log (resistin)	ESD	0	1	Ref.	-0.14±0.24	0.138	-0.09±0.37	0.923
	Group 1	0.10	1.25	0.076	0.05±0.24	0.737	0.03±0.26	>0.999
	Group 2	-0.07	0.85	0.430	-0.10±0.25	0.963	-0.18±0.11	0.098
Log (visfatin)	ESD	0	1	Ref.	-0.05±0.53	>0.999	0.07±0.75	>0.999
	Group 1	-0.07	0.86	0.640	-0.09±0.63	>0.999	0.17±0.55	0.349
	Group 2	-0.11	0.78	0.651	-0.19±0.31	0.638	-0.27±0.19	0.126

[1]The difference in the change of each variable during the follow-up period, compared to the control group in the group of gastrectomy with duodenal bypass (group 1) and the group of gastrectomy without duodenal bypass (group 2), is estimated using a linear mixed model. The estimates and p values are adjusted for age, sex, time from the baseline, and the baseline measurements of each assessed variable.

[2]The statistical significance of the change in each variable at each visit compared to the baseline is assessed using paired *t*-test. P values are adjusted using the method of Dunnett for multiple comparisons between two visit points and the baseline.

Significant values ($p<0.05$) are in boldface type.

SD, standard deviation; ESD, endoscopic submucosal dissection; Ref, reference value; BMI, body mass index; Log, log10-transformed; GIP, gastric inhibitory polypeptide; GLP-1, glucagon-like peptide-1; PAI-1, plasminogen activator inhibitor-1.

Supplementary Table S2. Changes in the effects of gastrectomy with duodenal bypass according to the candidate effect modifiers, on the probability of better 1-year glycemic control at 1-year visit.

Candidate effect modifiers	Estimates for the interaction terms	
	Changes in OR (95%CI)	p for interaction
BMI (per kg/m^2)	1.42 (0.86-2.35)	0.167
HbA1c (per %point)	0.49 (0.17-1.40)	0.182
Fasting glucose (per 10 mg/dL)	0.78 (0.46-1.33)	0.360
Postprandial 2-hour glucose (per 10 mg/dL)	0.94 (0.80-1.10)	0.420
HOMA-IR (per 1)	2.16 (0.75-6.21)	0.152
**Fasting**		
Ghrelin (per pg/mL)	1.00 (1.00-1.00)	0.978
GIP (per pg/mL)	1.00 (0.99-1.00)	0.356
GLP-1 (per pg/mL)	1.00 (0.99-1.01)	0.446
Glucagon (per pg/mL)	1.01 (1.00-1.02)	0.117
**Leptin (per ng/mL)**	**3.06 (1.29-7.27)**	**0.011**
**PAI-1 (per ng/mL)**	**0.95 (0.91-0.99)**	**0.013**
Resistin (per ng/mL)	1.17 (0.77-1.77)	0.463
Visfatin (per ng/mL)	1.09 (0.75-1.59)	0.655
**Postprandial 2 hours**		
Ghrelin (per pg/mL)	1.00 (1.00-1.00)	0.713
GIP (per pg/mL)	1.00 (0.99-1.01)	0.754
GLP-1 (per pg/mL)	1.00 (0.99-1.01)	0.436
Glucagon (per pg/mL)	1.01 (1.00-1.02)	0.125
**Leptin (per ng/mL)**	**3.67 (1.39-9.71)**	**0.009**
PAI-1 (per ng/mL)	0.99 (0.97-1.01)	0.358
Resistin (per ng/mL)	1.23 (0.74-2.03)	0.424
Visfatin (per ng/mL)	0.99 (0.66-1.49)	0.973

Changes in the effects of gastrectomy with duodenal bypass on 1-year glycemic control status (with endoscopic submucosal dissection as the control) are presented according to candidate

effect modifiers. Each interaction term was the product of 'gastrectomy with duodenal bypass' and the level of the candidate effect modifier. Significance of the effect modifications are tested by entering the interaction terms into the ordered logistic regression model for 1-year glycemic control status (the order of 'improved', 'equivocal', and 'worsened'). The estimates and p values are adjusted for ‘gastrectomy without duodenal bypass’ and HOMA-IR. Significant values (p for interaction <0.05) are in boldface type.

OR, odds ratio; CI, confidence interval; BMI, body mass index; HbA1c, hemoglobin A1c; HOMA-IR, homeostasis model assessment-insulin resistance; GIP, gastric inhibitory polypeptide; GLP-1, glucagon-like peptide-1; PAI-1, plasminogen activator inhibitor-1.

Supplementary figures legends

Supplementary Figure S1. Glycemic control status at 1-year visit according to the type of intervention. ESD, endoscopic submucosal dissection

Supplementary Figure S2. Changes in metabolic parameters from the baseline values.

(a) HbA1c, (b) fasting glucose, (c) postprandial glucose, (d) body mass index (BMI), (e) Homeostasis model of insulin resistance (HOMA-IR) during the follow-up period, according to the type of intervention.

§ $p<0.05$ vs. ESD group (adjusted for age, sex, time from the baseline, and the baseline measurements of each metabolic parameter)

[*] $p<0.05$ vs. baseline (adjusted for multiple comparisons using the method of Dunnett)

Supplementary Figure S3. Kaplan-Meier curves for composite event (recurrence of gastric cancer, myocardial infarction, stroke, coronary revascularization, and all cause death) according to the type of intervention.

Supplementary figures

Figure S1

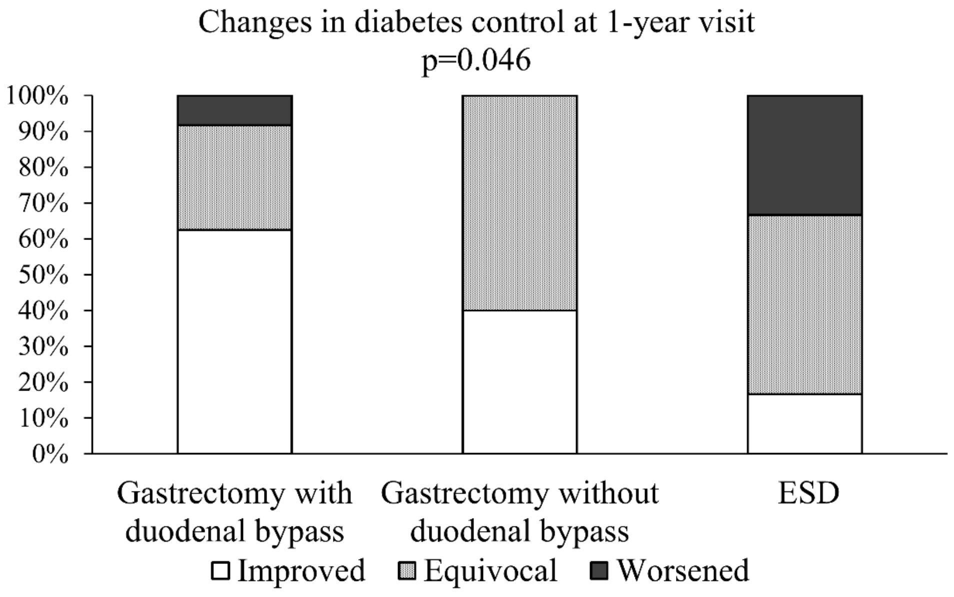

Figure S2

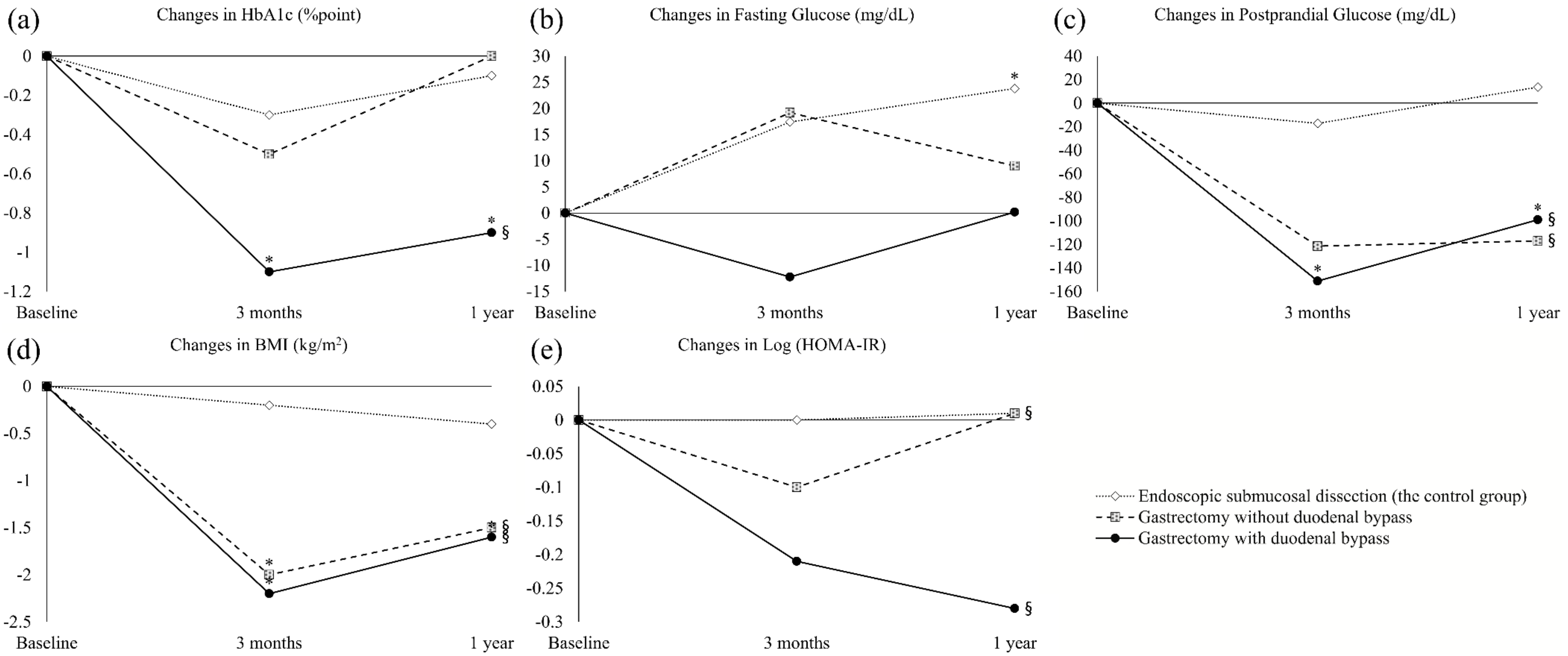
(a)
Changes in HbA1c (%point)
0
-0.2
-0.4
-0.6
-0.8
-1
-1.2
Baseline
3 months
1 year
*
*
§
(b)
Changes in Fasting Glucose (mg/dL)
30
25
20
15
10
5
0
-5
-10
-15
Baseline
3 months
1 year
*
(c)
Changes in Postprandial Glucose (mg/dL)
40
20
0
-20
-40
-60
-80
-100
-120
-140
-160
Baseline
3 months
1 year
*
*
§
§
(d)
Changes in BMI (kg/m2)
0
-0.5
-1
-1.5
-2
-2.5
Baseline
3 months
1 year
*
*
*
§
§
(e)
Changes in Log (HOMA-IR)
0.05
0
-0.05
-0.1
-0.15
-0.2
-0.25
-0.3
Baseline
3 months
1 year
§
§
Endoscopic submucosal dissection (the control group)
Gastrectomy without duodenal bypass
Gastrectomy with duodenal bypass

Figure S3

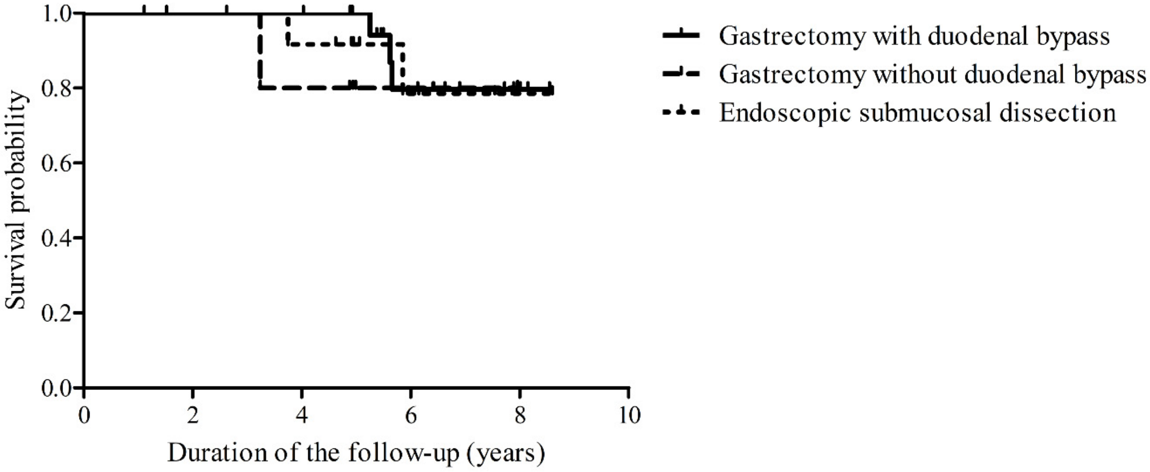
1.0
0.8
0.6
0.4
0.2
0.0
Survival probability
0
2
4
6
8
10
Duration of the follow-up (years)
Gastrectomy with duodenal bypass
Gastrectomy without duodenal bypass
Endoscopic submucosal dissection
